# Explaining the impact of mutations on quantification of SARS-CoV-2 in wastewater

**DOI:** 10.1038/s41598-024-62659-y

**Published:** 2024-05-30

**Authors:** Noriko Endo, Yoshiaki Nihei, Tomonori Fujita, Makoto Yasojima, Fumi Daigo, Hiroaki Takemori, Masafumi Nakamura, Ryo Matsuda, Sorn Sovannrlaksmy, Masaru Ihara

**Affiliations:** 1 Research Center for Environmental Quality Management, Graduate School of Engineering, Kyoto University, 1-2 Yumihama, Otsu, Shiga 520-0811 Japan; 2 Water Agency, Inc., 3-25 Higashi-Goken-cho, Shinjuku-ku, Tokyo, 162-0813 Japan; 3 Shimadzu Techno-Research, Inc., 1 Nishinokyo Shimoai-cho, Nakagyo-ku, Kyoto, 604-8436 Japan; 4 Hiyoshi Corporation, 908 Kitanosho, Omihachiman, Shiga 523-0806 Japan; 5 Faculty of Agriculture and Marine Science, Kochi University, 200 Monobe-Otsu, Nankoku City, Kochi 783-8502 Japan

**Keywords:** Environmental sciences, PCR-based techniques

## Abstract

Wastewater surveillance is an effective tool for monitoring community spread of COVID-19 and other diseases. Quantitative PCR (qPCR) analysis for wastewater surveillance is more susceptible to mutations in target genome regions than binary PCR analysis for clinical surveillance. The SARS-CoV-2 concentrations in wastewater estimated by N1 and N2 qPCR assays started to diverge around July 2022 in data from different sampling sites, analytical methods, and analytical laboratories in Japan. On the basis of clinical genomic surveillance data and experimental data, we demonstrate that the divergence is due to two mutations in the N1 probe region, which can cause underestimation of viral concentrations. We further show that this inaccuracy can be alleviated if the qPCR data are analyzed with the second derivative method or the Cy0 method instead of the crossing point method.

## Introduction

Wastewater surveillance has emerged as an effective tool for monitoring community spread of COVID-19 and other diseases and to complement public health systems. As a mixture of stools and urine from individuals with a connection to a sewer system, wastewater is an important, inexpensive, and equitable source of public health information. Many countries adopted wastewater surveillance during the COVID-19 pandemic^1^. Now, many of them operate wastewater surveillance systems for (future) pandemic preparedness and plan to continue it even after the expiration of the public health emergency declaration^2^. As the COVID-19 pandemic had many phases, wastewater surveillance has proven to be useful as an unbiased and representative way of monitoring community spread of COVID-19 that is independent of testing capacity and reporting requirements in the clinical surveillance system. However, interpretation of the quantitative results of wastewater surveillance—i.e. SARS-CoV-2 virus concentrations in wastewater—may not be straightforward, because the amount of the virus shed in the stool can vary depending on the individual, vaccine status, and virus variant^3–6^. Moreover, quantification itself can be affected by mutations in target primer and probe regions used in quantitative PCR (qPCR) analysis. Mutations can lead to mismatches with the sequences of primers and probes originally designed for wild-type sequences. As a result, the efficiency and accuracy of qPCR quantification may be compromised^7–9^.

The influence of viral genome mutations can be prominent in wastewater analysis by qPCR, but is less so in clinical testing by PCR, studies of which are much more common. As PCR primers and probes are designed to bind to specific genome sequences, mismatches due to mutations can reduce the efficacy of DNA and fluorescence amplification. In clinical testing by PCR, the endpoint fluorescence signals are read on a binary basis, and a minor attenuation of signals due to mutations has little effect on the results. On the other hand, in wastewater testing by qPCR, information on the growth of fluorescence amplification is used to determine a Cq (quantification cycle) value, which is then translated to a concentration. Attenuated fluorescence amplification signals can affect Cq values and therefore concentrations. Thus, genome mutations in target regions for qPCR analysis can influence quantification, reducing the accuracy of the analysis. However, not enough attention has been paid to this issue.

So far, few studies have investigated the effects of mutations on PCR analyses, and the reported effects are ambiguous. An earlier study reported that the C28311T mutation in the SARS-CoV-2 genome, which characterizes the omicron variant and lies in the US Centers for Disease Control and Prevention (CDC) N1 probe region, did not affect qPCR readings^10^. More recently, however, another study described cases where SARS-CoV-2 variants which contain both C28311T and A28330G mutations in the CDC N1 probe region caused a delay or even a complete failure of fluorescence amplification, causing a shift of Cq values or false negative results in clinical samples^11,12^. These different findings show that the significance of the influence of viral genome mutations in qPCR depends on the position and the type of mutations, as well as assays used for analysis. It merits further investigation.

**Figure 1:**
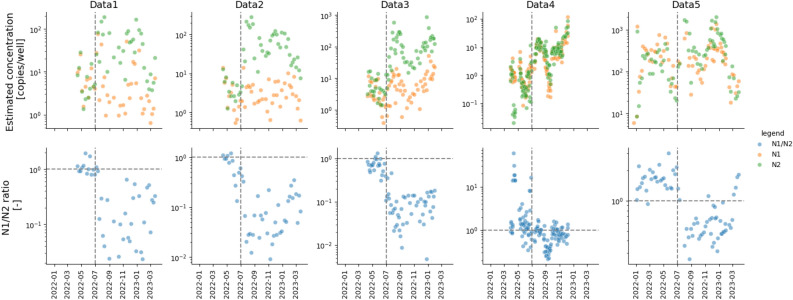
SARS-CoV-2 concentrations measured with N1 and N2 assays. (Top) Concentrations in wastewater as measured by the N1 assay (orange) and the N2 assay (green), in copies per well in Data 1–5. Concentrations were calculated by the crossing point (CP) method (more specifically the CP_manual method) to determine the Cq values for all datasets. (Bottom) Ratio between N1 and N2 concentrations (N1/N2; blue). The vertical dashed lines indicate 1 July 2022, the beginning of the 7th wave. The horizontal dashed lines were drawn at N1/N2 = 1, which means the two concentrations were equal.

During the second half of 2022, we noticed a discrepancy in SARS-CoV-2 concentrations in wastewater based on measurements from the CDC N1 and N2 regions, suggesting potential inaccuracies in at least one calculation. Moreover, the discrepancy started simultaneously across four wastewater treatment plants (WWTPs), three sample preparation methods, and three analytical labs in Japan (Table 1; Fig. 1). Subsequently, this study uncovered evidence indicating that genomic mutations in the CDC N1 probe region were responsible for this discrepancy.

**Table 1:** Summary of five observational data sets.

Data IDs	Data 1	Data 2	Data 3	Data 4	Data 5
Analytical labs	Lab A	Lab A	Lab A	Lab B	Lab C
Sampling locations	WWTP 1	WWTP 2	WWTP 3	WWTP 1	WWTP 4
Wastewater types	Influent to WWTP	Influent to WWTP	Influent to WWTP	Primary clarifier effluent (PE)	Influent to WWTP
Virus concentration methods	Solid precipitation	Solid precipitation	PEG precipitation	PEG precipitation	Direct capture
RNA extraction reagents	RNeasy PowerSoil Total RNA Kit (Qiagen)	RNeasy PowerSoil Total RNA Kit (Qiagen)	QIAamp Viral RNA Mini Kit (Qiagen)	QIAamp Viral RNA Mini Kit (Qiagen)	Maxwell RSC Enviro TNA Kit (Promega)
RT reagents	One Step PrimeScript RT-PCR Kit (Takara)	One Step PrimeScript RT-PCR Kit (Takara)	One Step PrimeScript RT-PCR Kit (Takara)	High-Capacity cDNA Reverse Transcription Kit (Applied Biosystems)	SARS-CoV2 RT-qPCR Detection Kit (Promega)
qPCR reagents				Gene Expression Master Mix (Applied Biosystems)	
Thermal cyclers	LightCycler 96 system (Roche)	LightCycler 96 system (Roche)	LightCycler 96 system (Roche)	Thermal Cycler Dice Real Time System III (Takara)	StepOnePlus Real-Time PCR System (Thermo Fisher Scientific)

Here we present (i) observations of the influence of SARS-CoV-2 genomic mutations on qPCR wastewater analysis, (ii) the hypothesis that the influence is due to mutations in the CDC N1 probe region, (iii) experimental results to illustrate the mechanism of the influence on qPCR analysis, and (iv) recommendations to reduce the influence of mutations and to improve the accuracy of analysis in a way that is easy to implement and applicable to both current and future mutations. This way, our aim is to raise awareness of the potential impacts of genomic mutations on virus quantification results using qPCR in wastewater surveillance. Additionally, we aim to provide recommendations for achieving more accurate quantitative analysis that is less susceptible to the influence of mutations.

## Results

### Observational wastewater surveillance data suggested that genomic mutations could lead to inaccurate estimation of virus concentrations by qPCR analysis

SARS-CoV-2 virus concentrations in wastewater estimated from the N1 and N2 assays by the crossing point (CP) method to determine Cq values started to diverge from the beginning of the 7th wave in July 2022 (Fig. 1). Until then, the ratio between the N1 and N2 concentrations (N1/N2) had been close to 1, but it declined significantly afterwards. The divergence was consistent across all five datasets (Data 1–5) acquired from four WWTPs by three sample preparation methods at three analytical laboratories (Table 1). In general, divergence was greatest at Lab A (Data 1–3). During the 7th wave, both Data 1 and Data 2 from the solid precipitation method and Data 3 from the PEG precipitation method resulted in 1 to 2 orders of magnitude smaller concentrations based on the N1 region than on the N2 region. Data 4 and Data 5 showed a similar though lesser trend. Noticeably, the magnitude of the N1/N2 divergence was not constant across the labs or sample preparation methods. However, the timing at which it started was almost identical across all of the datasets.

**Figure 2:**
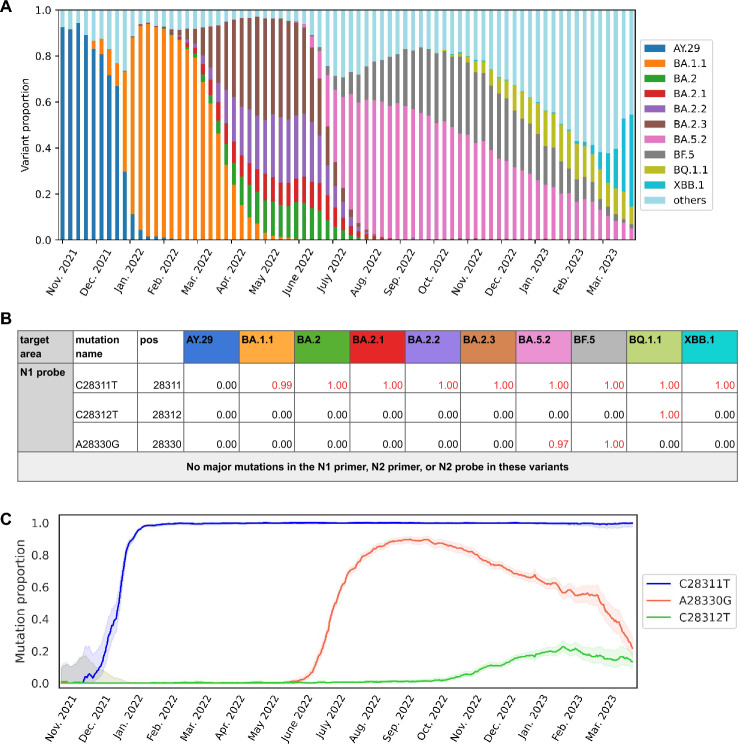
Dominant variants in Japan and their mutations in the N region. (**A**) Proportion of dominant SARS-CoV-2 variants that circulated in Japan from Nov 2021 through to Mar 2023. Data were obtained from the National Institute of Infectious Diseases, Japan. (**B**) Positions and types of mutations in the *N* region, and the fractions of these mutations in each of the dominant SARS-CoV-2 variants in Japan. The frequency (values > 0) of mutations in the N1 probe region is marked in red. No dominant variants had mutations in the N1 primer, N2 primer, or N2 probe region. Data were obtained from COVID CG (https://covidcg.org). (**C**) Temporal changes in the proportion of C28311T (blue), C28312T (green), and A28330G (red) mutations in clinical samples in Japan from Nov 2021 through to Mar 2023. Data were obtained from CoV-Spectrum (https://cov-spectrum.org/).

This divergence was likely attributable to a change in the fluorescence signals during the qPCR wastewater sample analysis. To investigate this, we calculated three indicators of the performance of the qPCR assays: endpoint fluorescence, maximum second derivative, and slope of the tangent. Endpoint fluorescence, as depicted in S. Fig. 1, indicates the efficiency of amplification or fluorescence throughout the thermal cycles. Maximum second derivative and slope of the tangent are relevant in performing the SDM and the Cy0 method, respectively. In all five data sets, all three indicators declined after July 2022 with the N1 assay, but changed little with the N2 assay (S. Fig. 2). These results, consistent across sites, methods, and labs, led to the hypothesis that genomic mutations in SARS-CoV-2 variants which emerged at the beginning of the 7th wave in Japan affected the performance of the qPCR analysis, particularly the N1 assay.

### Genomic surveillance data of clinical samples indicated introduction of variants with mutations in the N1 probe region

According to genomic surveillance data, dominant SARS-CoV-2 variants in Japan switched from delta sublineage AY. 29 to omicron sublineage BA.1.1 in the 6th wave (January–March 2022), to omicron sublineages BA.2.*x* in the 7th wave (July–September 2022), and then to omicron sublineages BA.5.2 and BF.5 in the 8th wave (October 2022–January 2023) (Fig. 2A). During this period, the major mutations present in the N1 and N2 genomic regions of the dominant variants circulating in Japan were C28311T, C28312T, and A28330G (Fig. 2B). All three mutations were located in the N1 probe region. No major variants that circulated in Japan during this period had significant mutations in the N1 primer region, the N2 primer region, or the N2 probe region. Delta sublineage AY.29 did not have any mutation in the N1 probe region (Fig. 2B). All omicron sublineages (BA1.1–XBB.1) have the C28311T mutation in the N1 probe region; sublineages BA.5.2 and BF.5 have double mutations (C28311T and A28330G), and BQ.1.1 also has double mutations (C28311T and C28312T) (Fig. 2B). Until the end of 2021, when AY.29 was dominant, no major mutations were present in the primer or probe regions of the N1 or N2 assay (Fig. 2). In January 2022, most SARS-CoV-2 viruses circulating in Japan had the C28311T mutation in the N1 probe region (Fig. 2C). After July 2022, both C28311T and A28330G appeared with BA.5.2 and BF.5. From around November 2022, C28312T appeared with the introduction of BQ.1.1.

### Experimental data demonstrated lowered intensity of fluorescence with mutations in the N1 assay

**Figure 3:**
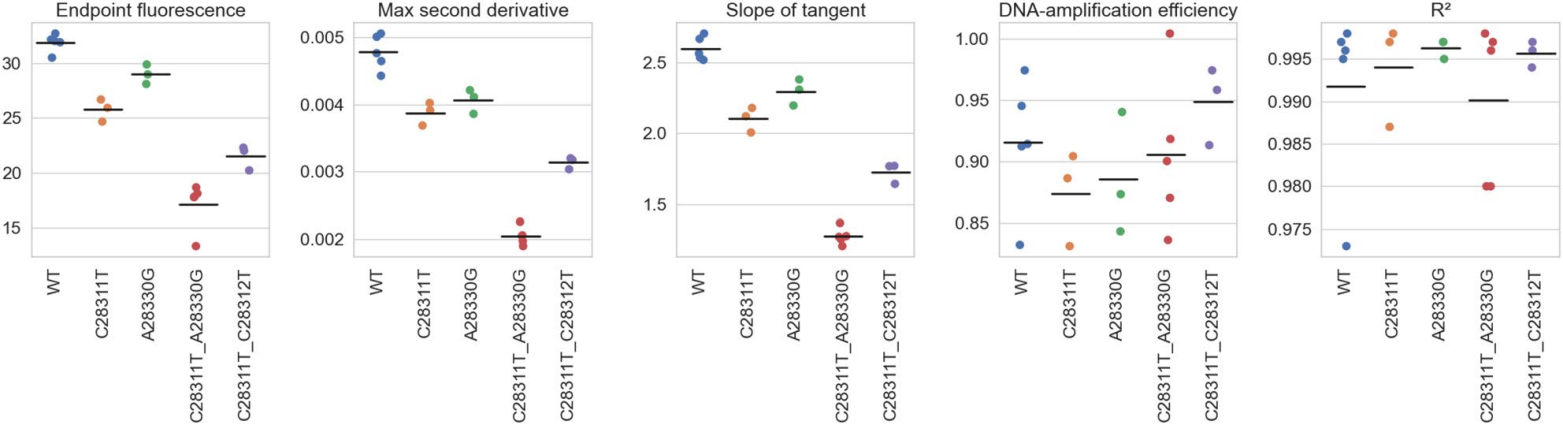
Performance of qPCR analysis with custom oligo DNAs with mismatches. Graphs show (L to R) endpoint fluorescence, maximum second derivative, the slope of tangent, DNA-amplification efficiency (calculated from the slope of the standard curve), and *R*^2^ of the standard curve. qPCR analysis was performed multiple times. Means are shown as: • within batches, — across batches (No. batches: 5 for WT, 3 for C28311T, 3 for A28330G, 5 for C28311T_A28330G, 3 for C28311T_C28312T).

We conducted qPCR analyses using custom oligo DNAs with the RT-qPCR assays at Lab B. The oligos contained the C28311T, C28312T, and A28330G mutations on the WT sequence, alone or in combination. In general, fluorescence signals were weaker with oligos with mutations than with those with the WT sequence (S. Fig. 3). To investigate the changes in detail, we analyzed five parameters of the fluorescence signal curves: endpoint fluorescence, maximum second derivative, slope of tangent, amplification efficiency, and *R*^2^ of the standard curve (Fig. 3).

The mean endpoint fluorescence values of C28311T (25.8) and A28330G (29.0) were significantly lower than that of WT (31.9) (Fig. 3; S. Table 1). Those of C28311T_C28312T (21.5) and C28311T_A28330G were even lower (17.1). Amplification efficiency and the slope of tangent showed similar trends. The declines of these three indicators were all statistically significant (S. Table 1). In general, these three parameters show that either the DNA-amplification efficiency by the primers or the fluorescence-reporting efficiency by the probe declined, without distinction.

As explained in Methods 5, DNA-amplification efficiency is a function of the slope of the standard curve and is independent of the fluorescence-reporting efficiency. The change of DNA-amplification efficiency was not significantly different among WT and the oligos with mutations (Fig. 3; S. Table 1), indicating that DNA-amplification efficiency by the primers was not compromised. This result is reasonable, because no mutations were located in the primer regions. *R*^2^ of the standard curve was high (> 0.98) in all cases and thus the variability of the analysis was small.

These results together show that the signal attenuation was due to the performance of the probe, and the effect was greatest when two mutations were present in the N1 probe region. Furthermore, C28311T + A28330G influenced performance more than C28311T + C28312T.

### Computational analysis finds some Cq calculation methods are less susceptible to the influence of mutations

**Figure 4:**
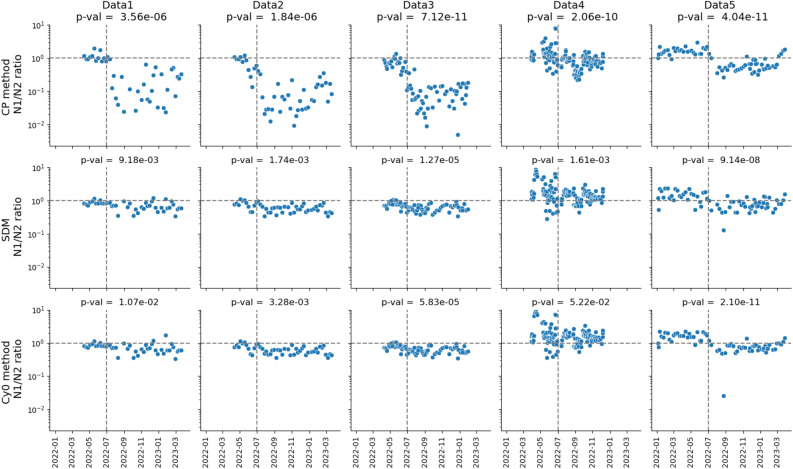
Time series of N1/N2 concentrations by the four Cq calculation methods. Rows indicate Cq calculation methods (top to bottom): CP_manual method, SDM, Cy0. Columns indicate data sets (L to R): Data 1–5. The horizontal dashed line shows where N1/N2 = 1. The vertical dashed line marks 1 July 2022. p-val is the associated *P*-value from the Mann–Whitney *U*-rank test, with the null hypothesis that N1/N2 follows the same distribution before and after 1 July 2022 against the two-sided alternative.

Given the effect of mutations on amplification curves in the qPCR analysis, we hypothesized that quantification using the crossing point (CP) method is especially susceptible to this effect, and that the second derivative method (SDM) or the Cy0 method (see "Methods" "Computational analysis of qPCR data") can reduce it (S. Figs. 1, 4). The CP method is the most common method used to calculate Cq values in qPCR analysis in Japan, and Data 1–5 (Fig. 1) were originally analyzed by it. Computational analysis demonstrated that the agreement of the N1 and N2 concentrations improved with the SDM and Cy0 methods relative to the CP method (the CP_manual method unless otherwise noted), though the degree of the improvement varied (Fig. 4, S. Table 2).

Analysis of Data 1–3 by Lab A with the SDM and the Cy0 method significantly improved the agreement of the N1 and N2 concentrations (S. Table 2). Although the Mann–Whitney *U*-rank test indicated that the distributions of N1/N2 before and after July 2022 still differed, the *P*-values indicated that they became closer with both the SDM (P = 9.18e–03) and the Cy0 method (P = 1.07e–02) as compared to the CP method (P = 3.56e–10) (Fig. 4). Analysis of Data 4 by Lab B using the CP method produced less N1/N2 separation than with Data 1–3 (S. Table 2), but the shift of N1/N2 was still significant (Mann–Whitney *U*-rank test, *P* = 2.06·e-10), and was again reduced with the SDM (*P* = 1.61e–03) and the Cy0 method (*P* = 5.22e–02). In analysis of Data 5 by Lab C, N1/N2 shifted from > 1 to < 1 around July 2022 in all Cq calculation methods, and the differences were still significant (CP, *P* = 4.04e–11; SDM, *P* = 9.14e–08; Cy0, *P* = 2.10.e–11; S. Table 2).

We also compared the performance of two forms of the CP method—CP_manual and CP_auto (see "Methods" "Computational analysis of qPCR data")—with Data 4 and Data 5, for which the CP_auto method was available (S. Table 2). The N1/N2 ratios before and after July 2022 were still significantly different (CP_auto, *P* = 2.25e–09 in Data 4, *P* = 1.15e–09 in Data 5). The CP_auto results were similar to those by the CP_manual method, suggesting that both methods are equally vulnerable to the effect of mutations.

## Discussion

Even though the WHO declared an end to the COVID-19 global health emergency in May 2023, we are not free of the risks of future pandemics. During the pandemic, wastewater surveillance was adopted to monitor SARS-CoV-2 and other infectious diseases for pandemic preparedness. To accurately assess new waves of infectious diseases, it is important that qPCR assays and analytical methods be robust and not influenced by mutations of pathogens.

Our results show that the introduction of BA.5.2 and BF.5 into Japan around July 2022 led to underestimation of the concentration of SARS-CoV-2 in wastewater when analyses were based on the CDC N1 region, as measured virus concentrations in wastewater began to diverge between the N1 and N2 assays at the beginning of the 7th wave (Fig. 1). This divergence was confirmed across three analytical labs, three methods, and four sampling locations in Japan, to varying degrees. During the 7th wave, the BA.5.2 and BF.5 variants became dominant, both of which had the C28311T and A28330G mutations in the N1 probe region (Fig. 2). Our experiments confirmed that those mutations decreased fluorescence signals in qPCR, which led to underestimation of the virus concentration when Cq values were calculated with the CP method. We concluded through genomic surveillance data and experimental data that the presence of both mutations together caused the significant underestimation of the N1-based concentration.

To our knowledge, this study is the first to comprehensively report the significance of genomic mutations in wastewater qPCR analysis. Indeed, Japan experienced the C28311T–A28330G double mutation most strongly in the world, possibly besides China (S. Fig. 5). Although the effect of the mutations on wastewater qPCR analysis could have been smaller in other countries, we expect that some level of underestimation was present if analyses were based on qPCR analysis with the CP method. This study also raises awareness of the potential effects of future mutations.

Underestimation of the N1-based SARS-CoV-2 concentration was especially prominent when the qPCR data were analyzed by the CP method. The fluorescence signals in qPCR grew more slowly with the sequences with mutations in the probe regions than with the wild-type sequence (S. Fig. 3). When the CP method is applied with a set threshold, the slow-growth fluorescence curves shift Cq values to the right (S. Fig. 4). Therefore, against standard curves for quantification established with the wild-type sequence, the delayed fluorescence curves evaluated with the CP method result in underestimation of virus concentrations.

We emphasize the importance of conducting qPCR analysis targeting multiple genomic regions, ideally for every sample or at least regularly. We first realized the issue by noticing the divergence of the concentrations estimated with the N1 and N2 regions. Although the absolute values of the estimated concentrations change over time with the spread of COVID-19 in communities, the ratio between the N1- and N2-based concentrations is expected to be constant at 1. It is thus helpful to monitor the N1/N2 ratio routinely to assess the validity of qPCR analysis results. The N1 region is currently the most commonly targeted around the world^1^, likely because the N1 assay was the most robust when wastewater surveillance was introduced early in the COVID-19 pandemic. Although N1-based analyses are often combined with analyses targeting the N2 or other regions, analyses do not always target multiple genomic regions^1^. In Japan, many municipalities conducted wastewater surveillance solely targeting the N1 region during a national pilot from July 2022 through to January 2023, partially owing to resource limitations^13^. Although it is possible to notice the issue of lowered efficiency with the N1 assay by using endpoint fluorescence or otherwise, we recommend to assess the reliability of qPCR analyses by comparing results from multiple targets.

The SDM and the Cy0 method can reduce the effect of mutations in the target genomic regions in the qPCR analysis relative to the CP method. While developing new qPCR assays to target new SARS-CoV-2 variants as they emerge might be one way to overcome the issue of mutation, this option is not ideal for this rapidly mutating virus. Developing qPCR assays with primers and probes for regions that are conserved is another way^14^, but this approach is also not immune to potential effects of future mutations. Anticipating future mutations and developing the best assays for those is unworkable. Therefore, addressing the issue analytically, accommodating and adjusting to the effects of mutations, offers a more robust and practical way for handling qPCR data. In addition, the SDM and the Cy0 method are more accurate when polymerization and fluorescence are inhibited^15^. Among those, the SDM is often equipped in qPCR instruments along with the CP method, and it is straightforward to apply the method with default software. It is worthwhile to consider using the SDM, especially when mutations are expected to exist in target genome regions.

The use of digital PCR (dPCR) can also improve the accuracy of quantification analysis in the presence of mutations. In general, dPCR is less susceptible to inhibition and is expected to be less susceptible to the effects of the mutations described here. While attenuation of fluorescence signals due to mutations does not affect quantification results per se in dPCR, Schussman et al.^16^ found an interesting application of the information of different endpoint fluorescence signals within individual droplets to track omicron variants accurately. Although dPCR is more expensive than conventional qPCR and is still less common, its advantage in wastewater surveillance needs to be recognized.

Our experiment demonstrates intriguing dynamics of the effect of mutations on qPCR analysis. With only one mutation in the probe region, the suppression of fluorescence signals was small. With two mutations, the suppression was more prominent, but the magnitude of the effect depended on the location of the mutations. When two mutations existed separately at positions 28 311 and 28 330, as in BA.5.2 and BF.5, the signal suppression was stronger than when two mutations occurred consecutively at 28 311 and 28 312, as in BQ.1.1. The effects of the number and location of mutations on qPCR analysis seem to be complex^7–9^. We also saw that the attenuation of fluorescence signals was greatest around September 2022 (S. Fig. 2), when BA.5.2 and BF.5 together were circulating at the highest proportion in Japan. BQ.1.1 crept in following that, and the signal attenuation became less significant, which aligns with the experimental results.

While this study provides compelling evidence that mutations in target genomic regions can reduce the accuracy of qPCR analysis, the significance of these effects varies. Notably, Data 4 and Data 5 exhibited less profound impacts compared to Data 1–3. This variance was likely attributed to the utilization of distinct RNA extraction reagents, RT reagents, qPCR reagents, and thermal cyclers for Data 4–5 as opposed to Data 1–3 (Table 1). Additionally, sample types might have had some influence, as Data 4 was the only sample where wastewater was collected from the primary effluent and experienced the least attenuation of the N1 signal. It is plausible that future variants could interact with assays and sample types differently from what we observed in this study. Consequently, conducting further systematic analysis and fostering global knowledge-sharing initiatives would benefit wastewater surveillance using qPCR analysis.

## Conclusions

Across multiple analytical labs, methods, and sampling locations, the estimates of SARS-CoV-2 concentrations in wastewater based on the CDC N1 and N2 regions started to diverge from around July 2022 in Japan. This timing coincided with the arrival of BA.5.2 and BF.5, which have two mutations in the N1 probe region. Our comprehensive experiments demonstrated that the mutations attenuated fluorescence signals in qPCR. This attenuation, combined with the CP method, increased the Cq values and thus lowered concentration estimates when the N1 region was targeted. We recommend using multiple targets in qPCR analysis to identify any potential effects of mutations, which can happen unknowingly with the introduction or emergence of new variants. We also recommend the use of the SDM or the Cy0 method instead of the CP method to reduce the effect of mutations on qPCR analysis for more accurate analysis.

## Materials and methods

### Wastewater surveillance data

We analyzed five datasets (Data 1–5) derived from wastewater surveillance data from three analytical labs, three sample preparation methods (virus concentration and RNA extraction), two wastewater types, and four wastewater treatment plants (WWTPs) in Japan (Table 1). Wastewater samples were collected from April 2022 through to the beginning of 2023 with some variations. The longest dataset (Data 5) spans from January 2022 through to March 2023 and covers the 6th wave in Japan (January 2022–March 2022), the 7th wave (July 2022–September 2022), and the 8th wave (October 2022–January 2023).

For Data 1 and Data 2, wastewater samples were collected from the influent to WWTPs 1 and 2, respectively, and analyzed by Lab A. The data were collected from April 2022 through to March 2023. The virus concentration was assayed by using the solid precipitation method^17^. In brief, 240 mL of WWTP influent was centrifuged at 3000×*g* for 5 min. The precipitate was applied to an RNeasy PowerSoil Total RNA Kit (Qiagen Inc., Hilden, Germany) to obtain an RNA extract of 100 µL. Then 5 µL of RNA extract was applied to a One Step PrimeScript RT-PCR Kit (Takara Bio Inc., Shiga, Japan), and SARS-CoV-2 RNA was quantified by both CDC N1 and N2 primer sets on a LightCycler 96 system (Roche Molecular Systems Inc., Switzerland). The thermal cycling conditions for both assays consisted of 42 °C for 5 min and 95 °C for 10 min, followed by 45 cycles of amplification at 95 °C for 5 s, and annealing and extension at 60 °C for 30 s. Each sample was measured in technical quadruplicate. A 1:10 serial dilution of oligo RNA (10^1^–10^5^ copies/well) including both N1 and N2 targets (Japan Gene Research Institute Co., Ltd., Shizuoka, Japan) was used to generate standard curves and as a positive control for the RT-qPCR assay. RNase-free water was used as a negative control.

For Data 3, wastewater samples were collected from the influent to WWTP 3 from April 2022 through to January 2023 and analyzed by Lab A. The virus concentration was assayed by PEG precipitation^18^. In brief, 240 mL of influent was centrifuged at 3000×*g* for 5 min, and the supernatant was transferred to a fresh centrifuge tube. Then PEG 8000 (molecular biology grade, average mol. wt. 8000; Sigma-Aldrich Co. LLC, St. Louis, MO, US) and NaCl were added to final concentrations of 10% (w/v) and 1 M, respectively. The samples were incubated at 4 °C overnight with gentle agitation. After centrifugation at 10 000 × *g* for 30 min, the PEG precipitate, containing the virus, was suspended in 500 µL of phosphate buffer solution (for biochemistry, 0.1 M, pH 8.0, FUJIFILM Wako Pure Chemical Industries, Ltd., Osaka, Japan) to give a total volume of ~ 700 µL. From 140 µL of the virus concentrate, RNA was extracted with a QIAamp Viral RNA Mini kit (Qiagen) as per the manufacturer’s instructions to obtain a 65-µL extract. In the RNA extraction step, PCR-grade water (Takara) was included as a negative control every time. Then 5 µL of RNA extract was applied to a One Step PrimeScript RT-PCR Kit (Takara), and SARS-CoV-2 RNA was quantified by both CDC N1 and N2 primer sets as described above. Each sample was measured in technical quadruplicate.

For Data 4, wastewater samples were collected from the primary clarifier effluent (PE) at WWTP 1 from April 2022 through to December 2022 and analyzed by Lab B. Virus in wastewater was concentrated by PEG precipitation method as previously described^19^. In brief, 120 mL of PE was centrifuged at 4500×*g* for 10 min, and the supernatant was transferred to a fresh centrifuge tube. Then PEG 8000 and NaCl were added to final concentrations of 10% (w/v) and 1 M. The samples were incubated at 4 °C overnight with gentle agitation. After centrifugation at 10 000×*g* for 30 min, the PEG precipitate, containing the virus, was dissolved in 500 µL of phosphate buffer solution (0.1 M, pH 8.0, Wako) to give a total volume of ~ 700 µL. From 140 µL of the virus concentrate, RNA was extracted with a QIAamp Viral RNA Mini kit to obtain an 80-µL extract. In the RNA extraction step, PCR-grade water (Roche) was included as a negative control every time. For reverse transcription (RT), a High-Capacity cDNA Reverse Transcription Kit (Applied Biosystems LLC, Foster City, CA, USA) was used to obtain 70 µL of cDNA from 35 µL of viral RNA as per the manufacturer’s protocol. For the RT step, PCR-grade water was included as a negative control every time. Five µL of template cDNA was applied, and the SARS-CoV-2 RNA was quantified by both CDC N1 and N2 primer sets in Gene Expression Master Mix (Applied Biosystems). qPCR was performed in a Thermal Cycler Dice Real Time System III (Takara). The thermal cycling conditions for both assays consisted of pre-heating at 50 °C for 2 min and pre-denaturation at 95 °C for 10 min, followed by 50 cycles of amplification at 95 °C for 15 s, and annealing and extension at 60 °C for 1 min. For each sample, qPCR was performed in a technical triplicate.

For Data 5, wastewater samples were collected from the influent to WWTP 4 from January 2022 through to March 2023 and analyzed by Lab C. Virus in the wastewater was concentrated by the direct capture method^20^. In brief, RNA was purified from 80 mL of the influent with a Maxwell RSC Enviro TNA kit (Promega Corp., Madison, WI, United States) as per the manufacturer’s protocol. From 500 µL of viral RNA solution, 5 µL was applied and the SARS-CoV-2 RNA was quantified by both CDC N1 and N2 primer sets in a SARS-CoV2 RT-qPCR Detection Kit (Promega). qPCR was performed in a StepOnePlus Real-Time PCR System (Thermo Fisher Scientific Inc., Waltham, MA, US). The thermal cycling conditions for both assays consisted of 45 °C for 15 min and 95 °C for 2 min, followed by 50 cycles of amplification at 95 °C for 3 s and 62 °C for 30 s. Each sample was measured in a technical triplicate.

### Clinical genomic surveillance data

Three sources of genomic surveillance data were used to identify the trends among the dominant SARS-CoV-2 variants in Japan and to identify their mutations. For the former objective, we used the nationwide genomic surveillance data of clinical specimens held by the National Institute of Infectious Diseases, Japan^21^. For the latter, we downloaded the global Lineage Reports data from COVID CG (https://covidcg.org)^22^ with the query “Mutation Type = NT, Consensus Threshold = 0.7.” We also used CoV-Spectrum (https://cov-spectrum.org/)^23^ to track the proportion of specific mutations in Japan. Both COVID CG and CoV-Spectrum are open resources for tracking circulation of SARS-CoV-2 variants and their genomic mutations, and the data are based on GISAID (https://gisaid.org/)^24^. We also used CoV-Spectrum to obtain the temporal trends of the proportions of the C28311T, C28312T, and A28330G mutations across countries.

### Experimental qPCR analyses using custom oligo DNAs with or without mutations

We conducted qPCR analyses using custom oligo DNAs including the SARS-CoV-2 sequence with or without mutations to investigate how the fluorescence signal curves were affected. Thermo Scientific synthesized the custom oligo DNAs including the CDC N1 region (positions 28,287–28,358) of the SARS-CoV-2 Wuhan-Hu-1 strain (GenBank Acc. No. MN908947.3, referred to here as the wild type, WT) and the WT sequence with the C28311T, A28330G, C28311T + A28330G (C28311T_A28330G), and C28311T + C28312T (C28311T_C28312T) mutations (S. Table 3). A 1:10 serial dilution of oligo DNAs was used to generate standard curves (10^1^–10^5^ copies/well). qPCR assays in 96-well plates were conducted in a 25-µL qPCR reaction volume containing 12.5 µL of Gene Expression Master Mix (Applied Biosystems), 1.0 µL of 10 µM forward and reverse primers (10 pmol each), 0.5 µL of 5 µM TaqMan probe (2.5 pmol), 5 µL of nuclease-free water, and 5 µL of oligo DNA. qPCR was performed in a Thermal Cycler Dice Real Time System III (Takara). We assessed the endpoint fluorescence, maximum second derivative, slope of tangent, and amplification efficiency of the fluorescence signal curves and *R*^2^ of the standard curve (see "Methods" "Computational analysis of qPCR data").

### Computational analysis of qPCR data

We used four methods to derive Cq values from qPCR fluorescence results: the crossing point (CP) manual threshold method (CP_manual method), the CP automatic threshold method (CP_auto method), the second derivative method (SDM), and the Cy0 method^15^ (S. Fig. 1). The CP method defines Cq as the PCR cycle number at which the fluorescence signal of the amplified product reaches a set threshold. The threshold can be set either manually by the user or automatically by the qPCR instrument. The SDM defines Cq as the PCR cycle number at which the second derivative of the fluorescence signal curve (also known as the amplification curve) takes the maximum. In the Cy0 method, Cq is calculated as the *x*-value (cycle number) of the intersection point between the *x*-axis and the tangent of the fluorescence signal curve at the inflection point^15^.

We calculated Cq values from normalized fluorescence data (also known as the primary curve or delta-*R*_n_) from the qPCR instruments. All of the qPCR instruments were equipped with the CP_manual method and the SDM, except for those in Lab A. None had the Cy0 method. Therefore, cycle-by-cycle normalized fluorescence data were exported from the instruments, and we applied the Cq calculation methods, except for the CP_auto method, offline in a script written in Python 3. The discrete cycle-by-cycle fluorescence data were fitted to the Richards equation so that derivatives and tangents could be calculated. Because the CP_auto method uses an instrument-derived threshold, Cq values were obtained only if the instruments had this method available. In the CP_manual method, thresholds were set at 0.05 for Data 1–3, 2 for Data 4, and 0.2 for Data 5. These thresholds were based on the region of exponential amplification and on values output by the CP_auto method from the respective thermal cyclers and on the manufacturers’ recommendations. Sample concentrations were evaluated with the four Cq calculation methods against the standard curve, against which the Cq values of WT cDNA templates were also calculated through the use of respective Cq calculation methods.

We assessed whether the results of the qPCR analyses were susceptible to the mutations from a time series of the ratio between the concentrations evaluated with the N1 and N2 regions (N1/N2). Under the assumption that the *N* genes in wastewater samples are intact and that the qPCR assays work at the same efficiency for the N1 and N2 regions, N1/N2 should hover at 1. Because the Kolmogorov–Smirnov test did not support that either N1/N2 or log-N1/N2 followed a normal distribution, we used the nonparametric Mann–Whitney *U*-rank test to investigate whether N1/N2 changed before and after 1 July 2022 with the two-sided alternative. We used the Python 3 scipy.stats sub-package for the statistical analyses.

### DNA-amplification efficiency and fluorescence-reporting efficiency

To discern the effect of primers and probes on qPCR, we defined *e* as “DNA-amplification efficiency” and *c* as “fluorescence-reporting efficiency.” Amplification efficiency is a commonly used parameter that defines the efficiency of DNA amplification^25^. At 100% efficiency (*e* = 1), DNA amplicons double in every PCR cycle. In practice, the amount of DNA amplification is judged from the strength of fluorescence, which is supposed to be a linear function of the amount of DNA amplified. In this sense, *e* determines the performance of the primers, and *c* determines the performance of the probes.

At cycle number *t* and the fluorescence strength of a reporter dye at successful prove cleavage being *f*_o_:

Amount of DNA amplicon: *N*_*t*_ = *N*_*t*−1_(1 + *e*) = *N*_0_(1 + *e*)^*t*^*.*

Strength of fluorescence: *F*_*t*_ = *c f*_o_ *N*_*t*−1_ = *c f*_o_ *N*_0_(1 + *e*)^*t-1*^*.*

In the CP method, the strength of fluorescence reaches a set threshold of $\underline{F}$ at *t* = Cq:

$$\underline {F} = c f_{o} N_{Cq - 1} = c f_{o} N_{0} \left( {1 + e} \right)^{Cq - 1} ,$$

$$\log (\underline {F} /cf_{o} ) \, = \, \log N_{0} + \, \left( {Cq \, - 1} \right) \, \log \left( {1 + e} \right),$$

$$Cq = \frac{log(\underline{F}/c {f}_{o})- log{ N}_{0}}{log(1 + e)}+1 = \left(\frac{\text{log}\left(\frac{\underline{F}}{c}{f}_{o}\right)}{\text{log}\left(1 + e\right)}+1\right)- \frac{1 }{log(1 + e)}log{N}_{0}$$

In a standard curve that defines the relationship between Cq and log*N*_0_, the slope is $-\frac{1}{log(1 + e)}$. This equation shows that the slope of a standard curve is solely a function of *e* and it is independent of *c*. On the flip side, the “DNA-amplification efficiency”, calculated as $e= {10}^{\frac{-1}{slope}}$, reflects only the efficiency of DNA amplification, indicating the performance of primers, but not the efficiency of fluorescence amplification or the performance of probes. The DNA-amplification efficiency used here follows this definition.

### Supplementary Information

Supplementary Information.

## Data Availability

The datasets generated and/or analysed during the current study are not publicly available because some the data are owned by the governments. However, upon reasonable request and with the permission of the respective data owners, the data can be made available from the corresponding author.
