# Supplementary material for: Explaining the impact of mutations on quantification of SARS-CoV-2 in wastewater
Source: Sci Rep. 2024 May 30;14:12482. doi: 10.1038/s41598-024-62659-y (PMC11139995; doi:10.1038/s41598-024-62659-y)
Supplement: Supplementary file 1 — Supplementary Information. [file 41598_2024_62659_MOESM1_ESM.docx]

Explaining the impact of mutations on quantification of SARS-CoV-2 in wastewater

Supplementary material


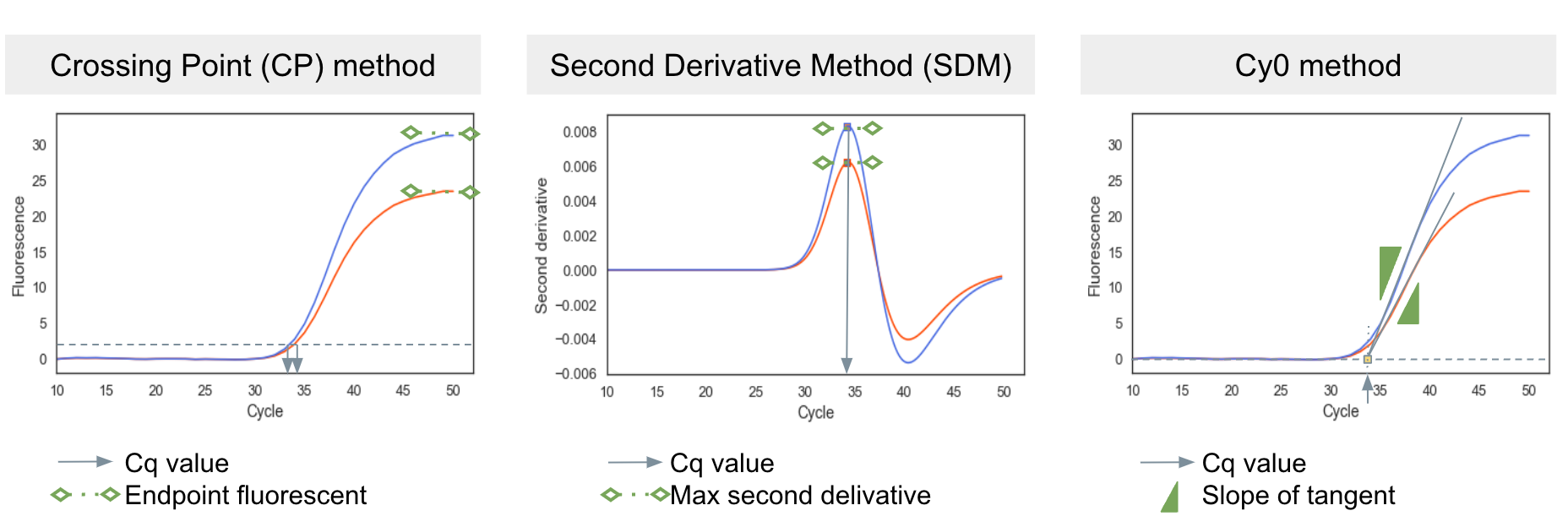


**S. Fig. 1: Conceptual illustrations of three indicators of qPCR performance and Cq calculation methods.**

The endpoint fluorescence (left) and the slope of tangent (right) are calculated from the qPCR fluorescence curve. The maximum second derivative (middle) is the peak of the second derivative of the curve. When fluorescence signals become weaker in samples (red curves) than normal (blue curves), the three qPCR performance indicators become smaller. In the CP method (left), the weaker fluorescence signals lead to a larger Cq value, which means that qPCR underestimates the virus concentration in wastewater. On the other hand, in principle, a Cq value determined by the second derivative method (SDM) (middle) or by the Cy0 method (right) is expected to be less or not affected even when the fluorescence signals become weak. For more about these methods, see “Methods” “Computational analysis of qPCR data” .


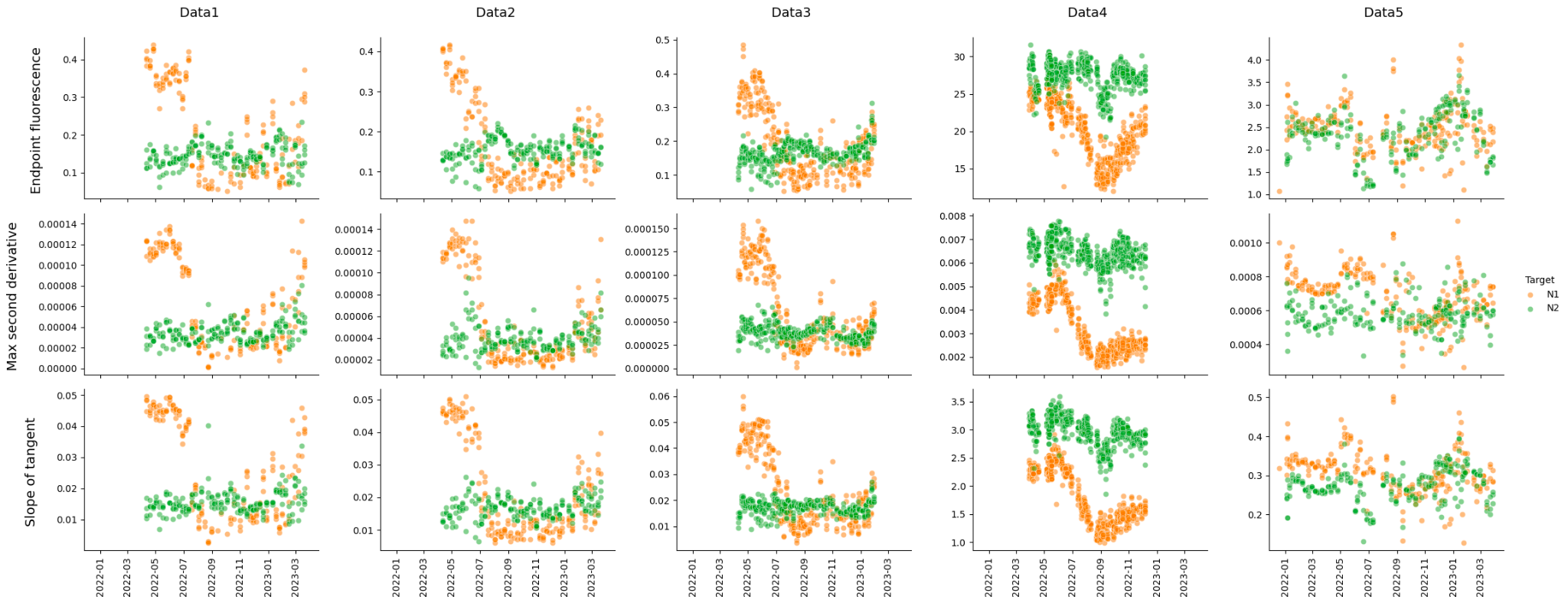


**S. Fig. 2: Three indicators of fluorescence signal attenuation.**

The endpoint fluorescence (top), the maximum second derivative (middle), and the slope of tangent (bottom) of Data 1–5 (each column) of the CDC N1 region (orange) and N2 region (green).


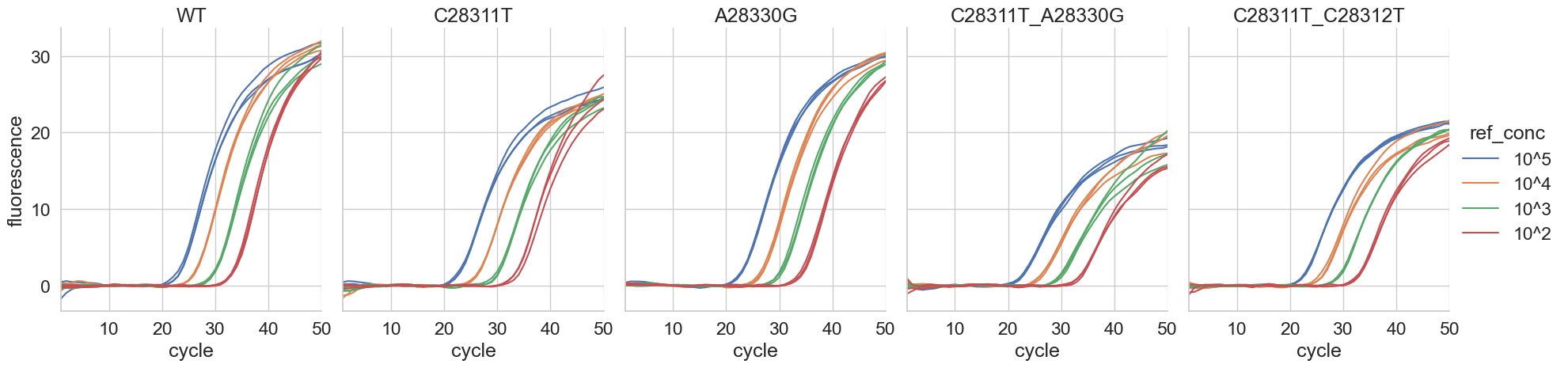


**S. Fig. 3: Fluorescence signal curves of the custom oligo DNAs with mismatches.**

Data obtained from CoV-Spectrum (<https://cov-spectrum.org/>) [23].


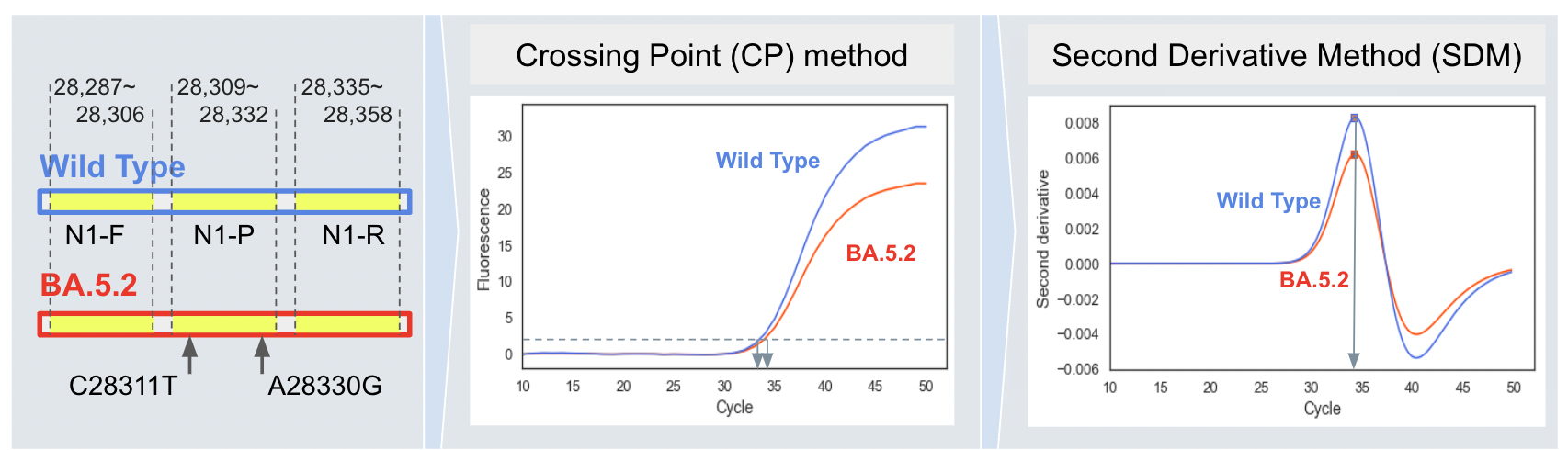


**S. Fig. 4: Conceptual description of the impact of genomic mutations on qPCR analysis using CP method and SDM.**

Omicron subvariant BA.5.2 has two mutations in the CDC N1 probe region. Compared with the amplification curve of the SARS-CoV-2 WT (blue), that of BA.5.2 (red) is attenuated. Therefore, the CP method shifts the Cq value to the right with BA.5.2. On the other hand, the Cq values are expected to be the same for the wild type and BA.5.2 by SDM.


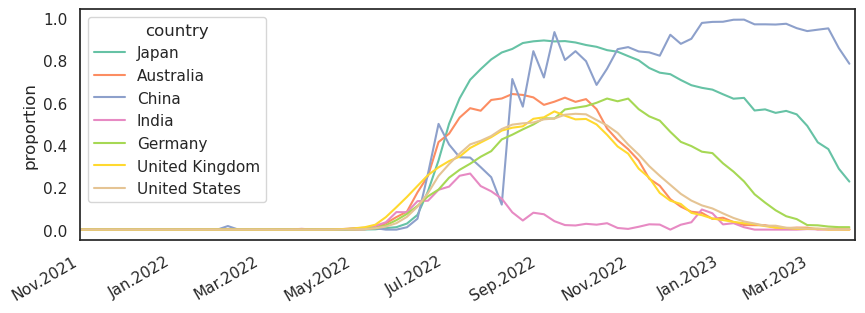


**S. Fig. 5: Multi-country comparison of the proportions of clinical samples with the C28311T–A28330G double mutation.**

**S. Table 1. Performance of qPCR analysis with custom oligo DNAs with mismatches.**

*P*-values were calculated from Student’s *t*-test under the hypothesis that the mean is the same as that of the wild-type sequence against the two-sided alternative. *Differences are statistically significant (*P* < 0.05).


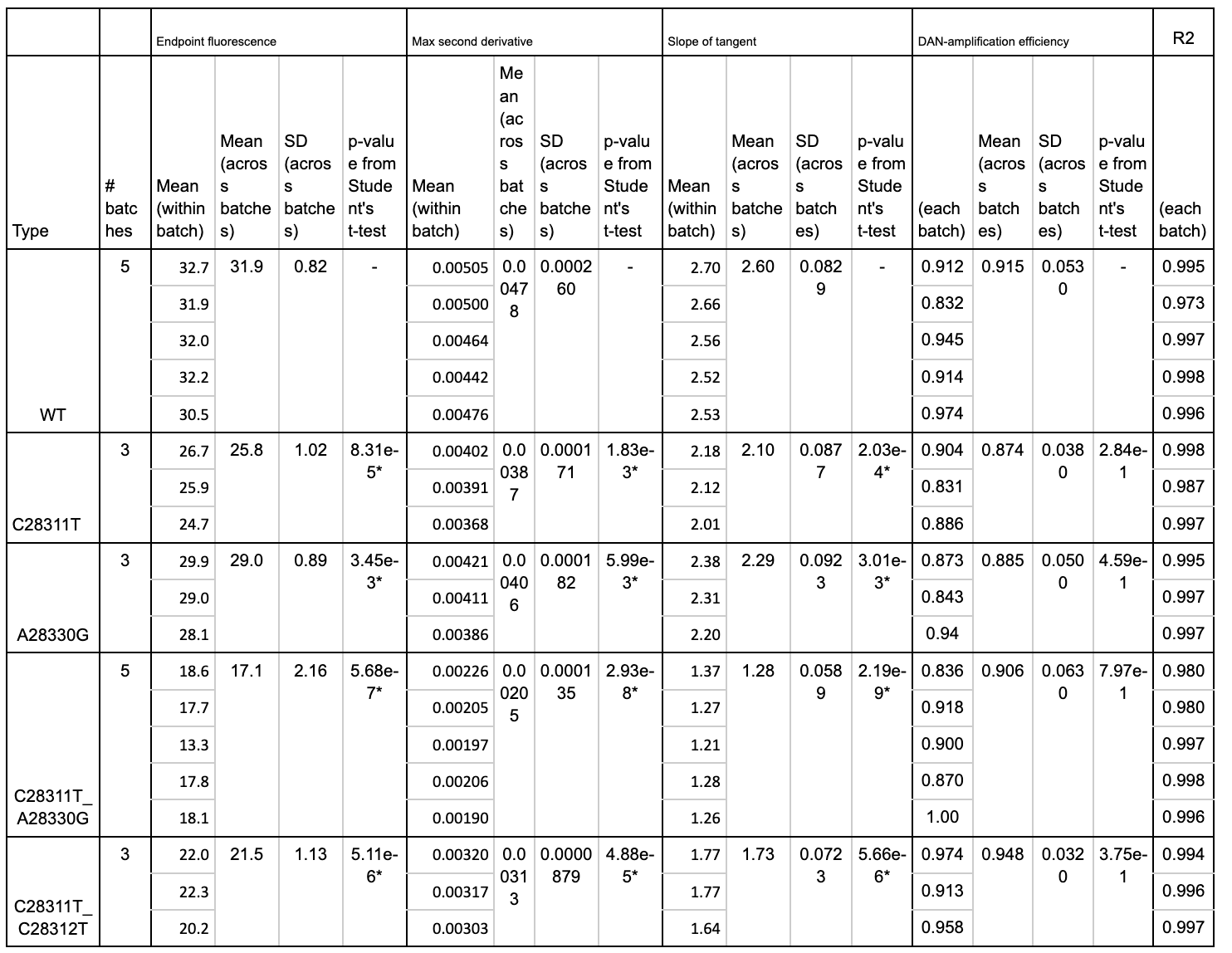


**S. Table 2: Median N1/N2 concentrations before and after 1 July 2022 by four Cq calculation methods.**

|  | Time | Data 1 | Data 2 | Data 3 | Data 4 | Data 5 |
| --- | --- | --- | --- | --- | --- | --- |
| Sample size (N) | before | 12 | 12 | 24 | 56 | 27 |
|  | after | 33 | 36 | 48 | 84 | 50 |
| conc_CP_manual | before | 0.997 | 0.824 | 0.718 | 1.38 | 1.65 |
|  | after | 0.120 | 0.0580 | 0.0838 | 0.795 | 0.583 |
| conc_CP_auto | before | NA | NA | NA | 1.18 | 1.49 |
|  | after | NA | NA | NA | 0.756 | 0.536 |
| conc_SDM | before | 0.823 | 0.794 | 0.776 | 1.79 | 1.37 |
|  | after | 0.647 | 0.572 | 0.583 | 1.34 | 0.715 |
| conc_Cy0 | before | 0.823 | 0.798 | 0.791 | 1.63 | 1.66 |
|  | after | 0.674 | 0.574 | 0.601 | 1.51 | 0.726 |

**S. Table 3: Sequence of the custom oligo DNAs**Within the SARS-CoV-2 N1 sequence (positions 28287–28358), the sequence of the 24 bp of the N1 probe region (28309–28332) is listed in this table. Mismatches with the wild type are shown in red.

| Custom oligo IDs | DNA sequence (the N1 probe region only) |
| --- | --- |
| WT | ACCCCGCATTACGTTTGGTGGACC |
| C28311T | ACTCCGCATTACGTTTGGTGGACC |
| A28330G | ACCCCGCATTACGTTTGGTGGGCC |
| C28311T_A28330G | ACTCCGCATTACGTTTGGTGGGCC |
| C28311T_C28312T | ACTTCGCATTACGTTTGGTGGACC |
